# Median arcuate ligament syndrome and aneurysm in the pancreaticoduodenal artery detected by retroperitoneal hemorrhage: A case report

**DOI:** 10.1002/ccr3.1643

**Published:** 2018-06-17

**Authors:** Takehiko Hanaki, Shiori Fukuta, Masaru Okamoto, Ayumi Tsuda, Takuki Yagyu, Shoichi Urushibara, Kanenori Endo, Kazunori Suzuki, Seiichi Nakamura, Masahide Ikeguchi

**Affiliations:** ^1^ Department of Surgery Tottori Prefectural Central Hospital Tottori Japan; ^2^ Department of General Internal Medicine Tottori Prefectural Central Hospital Tottori Japan

**Keywords:** celiac artery compression syndrome, median arcuate ligament syndrome, pancreaticoduodenal artery aneurysm, retroperitoneal hemorrhage

## Abstract

Here, we report a case with successful treatment of inferior pancreaticoduodenal artery aneurysm rupture due to celiac artery trunk compression caused by the median arcuate ligament. When clinicians see visceral aneurysms, the possibility of arcuate midline ligament compression syndrome (MALS) and ligamentectomy for MALS should be considered.

## 1 INTRODUCTION

Median arcuate ligament syndrome (MALS), also known as celiac artery/axis compression syndrome, is a rare cause of abdominal pain.^1^ MALS occurs due to extraluminal compression of the celiac artery (CA) by the median arcuate ligament (MAL), which is a part of the diaphragm. In this report, we describe the case of a 47‐year‐old male presenting with sudden epigastric pain diagnosed as aneurysm rupture in the inferior pancreaticoduodenal artery (PDA) and retroperitoneal hemorrhage. The patient was discharged with no major complications following emergent interventional radiology (IVR) for PDA coil embolization.

Decompression surgery was performed to prevent further aneurysm formation as the PDA aneurysm was thought to result from CA stenosis via extraluminal compression by MAL and increase blood flow from the SMA, which was developed to compensate for the CA perfusion area.

## 2 CASE PRESENTATION

**Figure 1:**
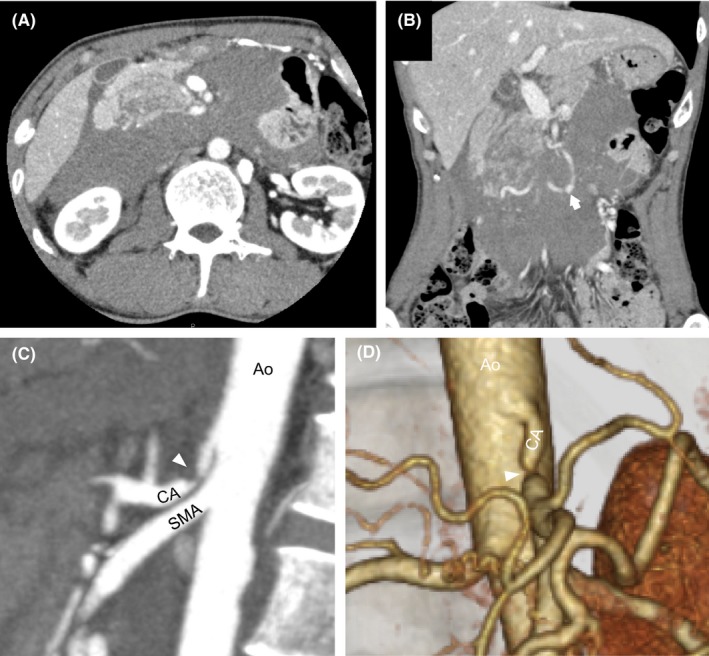
Contrast‐enhanced CT image shows retroperitoneal and intrapancreatic hematoma (A) and (B). Arrow shows aneurysm with extravasation in the pancreaticoduodenal artery (B). Sagittal arterial phase CT image reveals narrowing of the proximal celiac artery (arrowhead). Poststenotic dilatation is shown (C). Volume‐rendered 3D CT image shows narrowing of the celiac artery (arrowhead) caused by compression of the median arcuate ligament (D). Ao, aorta; CA, celiac artery; SMA, superior mesenteric artery

**Figure 2:**
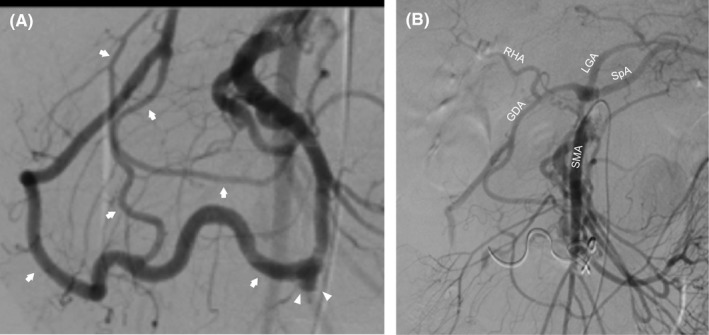
Digital subtraction angiography pre‐ (A) and post‐ (B) embolization Arrowheads show the aneurysm (diameter 9.3 mm) in the PDA, and several collateral arteries (arrows) are seen (A). Following embolization, superior mesenteric arteriography shows arterial enhancement in the area of celiac artery perfusion via collateral vessels (B). SMA, superior mesenteric artery; SpA, splenic artery; LGA, left gastric artery; RHA, right hepatic artery; GDA, gastroduodenal artery

The patient was a 47‐year‐old male presenting to our hospital with the chief complaint of sudden epigastric pain with vital signs indicating shock. One month earlier, he was hospitalized in our department and underwent omental patch repair for gastric ulcer perforation. Emergent contrast‐enhanced CT revealed a retroperitoneal hematoma in the pancreatic head (Figure 1A), aneurysm formation in the PDA (Figure 1B), and extraluminal compression to the celiac trunk (Figure 1C,D). Extravasation was also detected around the aneurysm in the PDA (Figure 2A), but no liquid was retained in the peritoneal cavity. Emergent IVR for hemostasis was performed for retroperitoneal hemorrhage from the ruptured PDA aneurysm. Arteriography of the superior mesenteric artery (SMA) was performed through a sheath introduced in the right femoral artery. Imaging of the SMA revealed the development of several collaterals in the pancreatic head (Figure 2A), and the common and proper hepatic arteries were imaged via these collaterals. The aneurysm in the PDA accompanied by leakage of the contrast‐enhanced agent had a diameter of 9.3 mm. IVR hemostasis was completed by PDA coil embolization. Subsequent SMA arteriography showed several collaterals other than embolized PDA perfusing the proper hepatic artery (Figure 2B). After IVR therapy, blood chemical analysis was monitored for several weeks, revealing that transaminase level was not elevated and hepatic infarction was considered negative. Obstruction of the duodenal passage resulting from the hematoma was observed for a few weeks; however, bypass surgery was not required as the obstruction was completely relieved as the hematoma was naturally and gradually absorbed.

**Figure 3:**
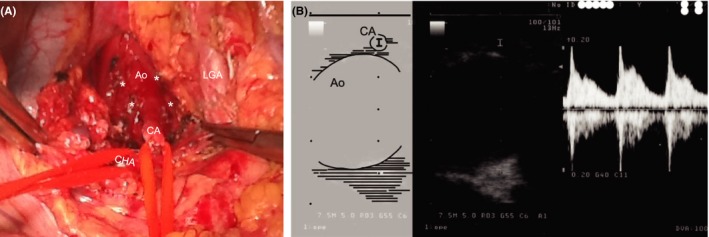
Intraoperative findings. The MAL(*) in front of the celiac artery is dissected (A). Ao, aorta; CA, celiac artery (taped); CHA, common hepatic artery (taped); LGA, left gastric artery. Intraoperative Doppler echo image after the MAL dissection (B). Sufficient arterial blood flow can be observed in the CA. Ao, aorta; CA, celiac artery

**Figure 4:**
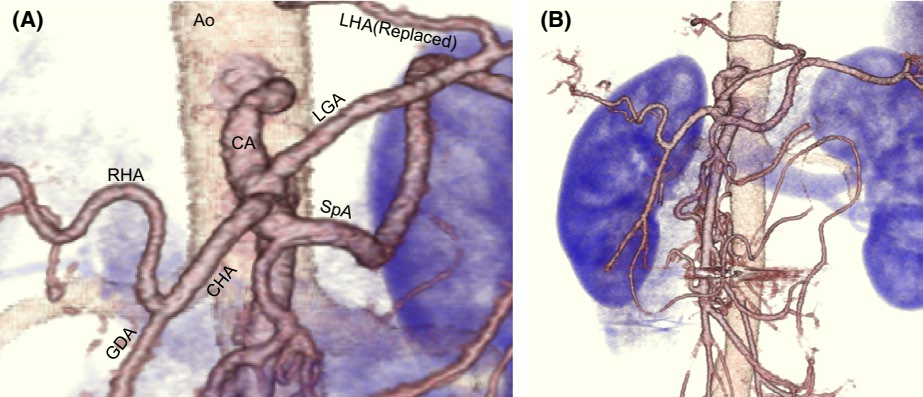
Enhanced CT (volume rendering) 6 mo after the operation. Celiac artery stenosis is not seen (A). Follow‐up CT shows no recurrence of the aneurysm (B). Ao, aorta; LHA, left hepatic artery; LGA, left gastric artery; CA, celiac artery; RHA, right hepatic artery; CHA, common hepatic artery; GDA, gastroduodenal artery; SpA, splenic artery

In this case, collateral perfusion pathways from the SMA developed to compensate for the reduced CA blood flow due to MAL compression, and the aneurysm might have generated in the PDA due to the abnormal changes in hemodynamic flow. Preservative treatment did not appear to relieve the CA compression. In addition, we considered that new aneurysms may form following new collateral circulation after IVR hemostasis. Therefore, incision of the MAL was necessary to release extraluminal compression of the CA. The surgery was performed 3 months following IVR hemostasis. Attributable to the patient's past medical history of peritonitis due to perforation of the gastric ulcer and the upper abdominal operation, we decided to perform conventional laparotomy instead of laparoscopic surgery. The laparotomy was performed with a midline abdominal incision, and the wall adhesion to the abdominal organs was peeled off, the lesser omentum incised, and the aortic fissure of the diaphragm exposed. In addition, the MAL on the anterior surface of the CA forming the aortic fissure was incised after taping the left and common hepatic arteries, respectively, (Figure 3A). The abdomen was closed after confirming that anterograde blood flow from the CA was restored with intraoperative Doppler ultrasonography (Figure 3B). The patient was discharged 8 days after the surgery with no complications. Follow‐up CT 6 months after the surgery confirmed the absence of stenosis in the CA with no de novo aneurysms (Figure 4A,B).

## 3 DISCUSSION

The MAL consists of the arcuate ligament and diaphragmatic crura, which forms an attachment from the diaphragm to the vertebrae. MAL connects the diaphragmatic crura crossing the aorta superior to the CA at the level of the first lumbar vertebrae.^2^ Occasionally, MAL is low lying, and thus it crosses the proximal portion of the CA. In 1917, Lipshutz first described MAL compression of the CA from an anatomical point of view.^3^ The symptoms of MAL compression of the CA trunk are known as MALS, also called celiac artery compression syndrome (CACS), and were first reported in 1963 by Harjola and in 1965 by Dunbar.^4^, ^5^

Midline ligament compression syndrome is a rare disease that occurs in 0.002% of the population^6^ and is characterized by weight loss, postprandial epigastric pain, nausea, and vomiting.^7^ As a result of the developments in imaging equipment in recent years, the frequency of identifying CA compression by MAL has increased. Symptoms accompanying MAL vary widely^1^ and it is asymptomatic in many cases.^8^

Aneurysms of the abdominal visceral arteries are relatively rare.^9^ The disease becomes problematic because it can rupture and follow a lethal clinical course, eg, in cases of hemorrhage in the peritoneal cavity, retroperitoneal space, gastrointestinal tract, and the biliary tract. Although detection frequency of aneurysms of the abdominal visceral arteries has increased recently due to the widespread use of CT angiography,^10^ aneurysms in the PDA are still rare and account for 2% of all visceral artery aneurysms.^9^ The PDA feeds the pancreas and duodenum and also forms an arterial arcade between the area of CA perfusion and the SMA perfusion area in the pancreatic head. An aneurysm in the PDA can be caused by atherosclerosis, celiac axis stenosis, pancreatitis, mycotic and bacterial infection, trauma, or fibromuscular hyperplasia.^9^, ^11^, ^12^, ^13^ Recent reports have linked MALS to PDA aneurysm.^14^, ^15^, ^16^

Some authors have described hemodynamic changes in aneurysm formation due to increased blood flow in the collateral arteries from the SMA to the CA perfusion area, and that increased arterial wall shear stress is responsible for aneurysm initiation, growth, and rupture.^8^, ^14^ Although there are several reported cases of aneurysm regression or stability following CA trunk reconstruction with stent placement, MAL incision, or bypass placement for the CA perfusion area,^16^, ^17^, ^18^ the mortality rate after rupture is still high, and even an unruptured small aneurysm is at risk of rupture, and regardless of the size, treatment for PDA aneurysm is recommended.^19^, ^20^ The ideal treatment for PDA aneurysm is currently considered to be IVR aneurysm embolization for both ruptured and unruptured cases if time and resources allow because of the high mortality rate in past open aneurysm resection surgeries and increased safety of IVR embolization in recent years.^19^, ^20^, ^21^ Intravascular treatment such as stenting for CA stenosis may help retrieve blood flow to the CA; however, since the cause of the stenosis is extraluminal compression and there are reports of restenosis after stenting,^7^ we believe that the standard therapy for MALS should be MAL incision surgery. In our case, to reduce the risk of new aneurysm formation and restore the appropriate CA blood flow, we decided to release the MAL using an open approach because the patient had a history of peritonitis a few months prior to the MAL incision surgery and intra‐abdominal adhesion was anticipated. Our case demonstrated normalized blood flow in the arterial arcade in the pancreatic head following successful treatment of the MAL incision (Figure 2A,B). However, laparoscopic MAL incision has recently been reported.^2^, ^7^ Although there is no long‐term prognostic report on MAL incision using laparoscopy compared with the open approach, technological innovations in laparoscopic surgery have been remarkable and have the added benefit of being minimally invasive,^15^ implying that the laparoscopic approach for MALS is useful and will be generalized in the near future.

In conclusion, we report a rare case of MALS caused by PDA aneurysm rupture and retroperitoneal hemorrhage. Blood flow to the arterial arcade in the pancreatic head from the SMA to the area of CA perfusion might be the cause of this rare and exceptional aneurysm formation in the PDA. Although there is no consensus on the necessity of surgical intervention for MAL compression of the CA, the present case suggests a potential benefit for operative MAL incision to decrease the abnormal blood flow causing shear stress in the arterial arcade in the pancreatic head, which is a possible cause for aneurysm formation.

## CONSENT

Written informed consent was obtained from the patient for publication of this case report and accompanying images. A copy of the written consent is available for review by the Editor‐in‐Chief of this journal on request.

## AUTHORSHIP

HT: gathered the patient data, performed a literature review, and wrote the manuscript. FS and OM: are the referring physician and provided clinical data. TA, YT, US, EK, and SK: revised the manuscript. NS and IM: were involved in overall supervision of the paper. All authors read and approved the final manuscript.

## CONFLICT OF INTEREST

The authors declare that they have no conflicts of interest.
